# Synthesis, Characterization, and Evaluation of a Novel Molecularly Imprinted Polymer (MIP) for Selective Quantification of Curcumin in Real Food Sample by UV-Vis Spectrophotometry

**DOI:** 10.3390/polym15163332

**Published:** 2023-08-08

**Authors:** Sergio Espinoza-Torres, Rosario López, Maria D. P. T. Sotomayor, Juan C. Tuesta, Gino Picasso, Sabir Khan

**Affiliations:** 1 Technology of Materials for Environmental Remediation Group (TecMARA), Faculty of Sciences, National University of Engineering, Av. Tupac Amaru 210, Rimac 15333, Peru; sespinozat@uni.pe (S.E.-T.); gpicasso@uni.edu.pe (G.P.); 2 Chemistry Institute, São Paulo State University (UNESP), Araraquara 14801-900, Brazil; m.sotomayor@unesp.br; 3 Laboratorio de Biotecnología, Universidad Nacional Autónoma de Alto Amazonas, Calle Prolongación Libertad 1220, Yurimaguas 16501, Peru; 4 Department of Natural Sciences, Mathematics, and Statistics, Federal Rural University of the Semi-Arid, Mossoro 59625-900, Brazil

**Keywords:** curcumin, MIP, bulk polymerization, adsorption

## Abstract

Curcumin is the main colorant of the curcuma longa plant, a food with many benefits for human health. This work aims to synthesize a novel molecularly imprinted polymer (MIP) for the selective detection of curcumin in real samples obtained from the local market of Peru. MIPs were synthesized via bulk polymerization using curcumin, acrylamide, ethylene glycol dimethacrylate, ABCV, and acetonitrile. FTIR spectra showed equal spectra for MIP and NIP. N_2_ physisorption analysis presented a higher value BET surface for the MIP (28.5 m^2^ g^−1^) compared to the NIP (18.5 m^2^ g^−1^). The adsorption capacity of the MIP was evaluated using UV-vis spectrophotometry in the band around 430 nm. The adsorption kinetics found were of pseudo-second-order and a Q_e_ value of 16.2 mg g^−1^. Furthermore, the adsorption process resembles the Freundlich adsorption model with a heterogeneity factor of less than 1 (0.61) and Kf greater for MIP (1.97). The selectivity test indicated that MIP is more selective for curcumin (Q = 13.20 mg g^−1^) than against interferents (Q = 2.19 mg g^−1^). The specific selectivity factor (S) obtained for the interferents was greater than 1 which indicates a good selectivity. Finally, the application of MIP in real samples using UV-vis spectrophotometry yielded a recovery value greater than 70%.

## 1. Introduction

Curcumin is the main colorant of the Curcuma longa plant [1], a food that has many benefits for human health, such as its anti-inflammatory, anticancer, antiviral, antioxidant, antibacterial, antidiabetic properties, and its ability to decrease the effects of COVID-19 [2,3], as well as its antiarthritic, antiatherosclerotic, antidepressant, and antiaging properties [4]. Curcumin also has great benefits in the treatment of Alzheimer’s [5]. Its beneficial properties have made it widely consumed in the food industry in ginger drinks, ginger spices, and curry powder [6] and in the pharmaceutical and cosmetic industry [7]. However, curcumin is often adulterated with other highly toxic dyes such as methanyl yellow [8] and lead chromate (II) [9]. Therefore, various detection and quantification methods are employed, such as HPLC [10,11], UV-visible spectrophotometry [12,13], fluorescence [14,15], electrochemistry [16], etc. The most widely used method is UV-visible spectrophotometry due to its simplicity, high efficiency, and good reliability. However, due to the low concentration of curcumin in foods [17] the use of UV-visible spectrophotometry is difficult due to the complexity of the previous treatment in the complex matrices of these foods [18]. Added to its low solubility in water, but solubility in organic solvents such as ethanol and acetone, it is insoluble in acid and neutral pH and slightly soluble in the basic or extremely acid medium [19], this suggests us to be more careful with the treatment and analysis of this dye. Due to this complication, the development of synthetic recognition materials capable of binding to specific molecules for their detection or removal from complex matrices is necessary; one of them is a molecular imprinting polymer (MIP). 

The selectivity of these materials is attributed to the specific cavities corresponding to the analyte that is synthesized. Its interaction can be compared to biological systems between antigen–antibody where the analyte is considered as the antigen and the MIP is considered as the antibody [20]. Imprinting polymers have shown great potential due to their low cost, speed and ease of synthesis, ability to selectively recognize various molecules, and a variety of applications for the detection of different analytes such as drugs [21,22,23,24], textile dyes [25,26], food dyes [27,28], and food [29,30]. Its main advantages are selective adsorption, high structural stability, robustness under various environmental and chemical conditions, and resistance to degradation, being able to be stored for years without losing efficiency [31,32,33]. 

Recent research focuses on increasing the detection capacity of MIPs, highlighting their immobilization on transducing surfaces such as fiber optics for chemical sensors or the MIP-optode [20,34]. MIP-based electrochemical sensors also exhibit excellent detection capability towards a wide range of target analytes [35,36]. Another advantage is the design of nano-MIPs, spherical nanoparticle MIPs with increased specific surface area, high impression factor, and many binding sites [37,38].

The present study focused on determining curcumin through the application of a curcumin-selective molecularly imprinted polymer (MIP) synthetized via bulk polymerization [39], in addition to showing the way for future applications in other sensor platforms in order to improve their selectivity properties. This synthesis method allows us to design the polymer’s structure to interact effectively with the curcumin molecule and enables the production of more uniform polymers with a high adsorption capacity. Due to its adsorption capacity and selectivity, it was successfully applied to determine curcumin in real food samples (Table 1).

## 2. Materials and Methods

### 2.1. Chemicals

Curcumin from curcuma longa (Turmeric) powder (C_21_H_20_O_6_, ≥66.5% (HPLC), for assay), acrylamide (C_3_H_5_NO, ≥99%, for electrophoresis), ethylene glycol dimethacrylate (EGDMA, C_10_H_14_O_4_, 98%), and 4,4′-Azobis(4-cyanovaleric acid) (ABCVA, HOCOCH_2_CH_2_C(CH_3_)(CN)N=NC(CH_3_)(CN)CH_2_CH_2_COOH, ≥98.0%) were purchased from Sigma Aldrich, St. Louis, MO, USA and ethanol absolute (EtOH, CH_3_CH_2_OH, for analysis EMPARTA^®^ ACS), methanol (MeOH, CH_3_OH, for liquid chromatography LiChrosolv^®^), and acetic acid (glacial) (CH_3_COOH, 100%) were purchased from Merck, Supelco, Milwaukee, WI, USA. All solutions were prepared in deionized water (18 MΩ cm at 25 °C), acquired from Milli Q Direct-0.3 purifier (Millipore). Interferents ivermectin (≥90%) sunset yellow (90%), tartrazine (≥85%), and acid blue 29 (≥40%) were purchased from Sigma Aldrich.

Samples of turmeric root and seasoning were obtained from a local market in Lima, Peru.

### 2.2. Synthesis of a Molecularly Imprinted Polymer by Bulk Polymerization

**Figure 1:**
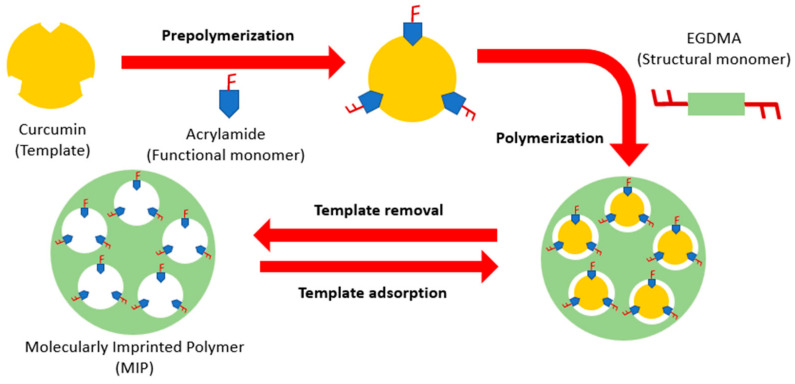
Molecularly imprinted polymer (MIP) synthesis diagram.

The synthesis of MIP (Figure 1) was carried out in a sealed flask initially containing curcumin (template) acrylamide (functional monomer) and acetonitrile (porogenic solvent). This mixture was stirred for 2 h to allow interaction between curcumin and the functional monomer. Then, the mixture was bubbled with N_2_(g) for 10 min. Subsequently, EGDMA (structural monomer) was added and bubbled again with N_2_(g) for 10 more minutes. ABCVA (radical initiator) was added and immediately it was placed in a water bath at 70 °C for 2 h. The molar ratio of the template, functional monomer, and structural monomer was 1:4:50 respectively, this molar ratio was chosen after assays described on Table S1. Non molecularly imprinted polymer (NIP) was prepared using the mentioned procedure but without adding curcumin.

Finally, curcumin was removed from the previously synthesized polymer (MIP). The extraction of curcumin was performed using a Soxhlet extraction system with mixtures of methanol and acetic acid glacial (90:10 and 70:30 *v*/*v*) for 72 h.

Spectroscopy UV-vis was applied to ensure that all the curcumin molecules (templates) had been removed from the supernatant. Finally, MIP and NIP were dried at 60 °C and then sieved to obtain particles of homogeneous size.

### 2.3. Characterization Experiments

The identification of various functional groups in the structure of the polymers or precursors was resolved using FTIR-Vertex 70 Spectrometer Bruker Shimadzu by absorbing infrared radiation from the bonds of the molecule of interest [43]. Different functional groups absorb radiation in different wavenumber ranges, giving rise to characteristic spectral peaks, described in graphs of transmittance (%) vs. wavenumber (cm^−1^) [44]. Surface analysis was performed using Micromeritics Gemini VII 2390, this procedure was carried out in two steps: (i) Degassing of MIP and NIP polymers using Helium gas at 80 °C for 2 h to remove any contaminants or particles from their surface; (ii) N_2_ adsorption measurements at 77K (liquid nitrogen temperature).

We obtain information on the specific surface area, average pore size, types of adsorption isotherms, and adsorption–desorption processes through the methods Barrett-Joyner-Halenda (BJH) and Brunauer-Emmett-Teller (BET). 

### 2.4. Adsorption Analysis of Curcumin and Optimization

**Figure 2:**
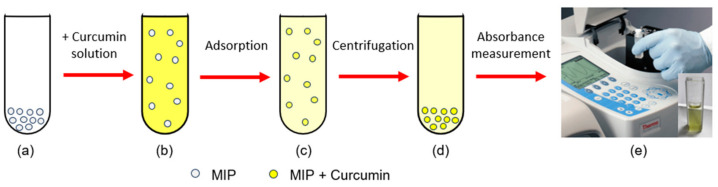
Stages of the adsorption process: (**a**) MIP, (**b**) Initiation of MIP-curcumin interaction; (**c**) End of MIP-curcumin interaction; (**d**) Separation of MIP from the solution by centrifugation; (**e**) Absorbance measurement of the remaining solution.

For the evaluation of the performance of the synthesized MIP against the curcumin dye (Figure 2), adsorption isotherms are performed, these isotherms represent the amount of an analyte adsorbed by an adsorbent surface (Q) based on parameters such as: mass, concentration, time, and adsorption medium. Equation (1) presents adsorption capacity (Q) in function of initial concentration (C_i_), final concentration (C_f_), absorbate volume (V), and polymer mass (m).

(1) $$Q=\frac{\left(C_{i}-C_{f}\right)\times V}{m}$$

### 2.5. Selectivity

Selectivity is evaluated by adsorption assays using different compounds called interferents; these can be other dyes, drugs, or compounds with a similar or different structure to the template. For this, it is necessary to know the parameters of selectivity and the impression factor. 

The first parameter is the partition coefficient of the adsorbate (K_P_), this relates the concentration of compound per gram of polymer (Q/mg g^−1^) and final concentrations of the template or interferent (C/mg mL^−1^) by means of the following equation:

(2) $$K_{P}=\frac{Q}{C}$$

On the other hand, the separation factor (α) refers to the selectivity of one molecule over another. It is calculated using the ratio of the partition coefficients (K_p_) of the template (curcumin) and an interferent for both the MIP and the NIP [45], following the equation

(3) $$\alpha =\frac{K_{p(CUR/MIP)}}{K_{p(INT/MIP)}}$$

Another important parameter is the impression factor (I); this factor compares the impression effects of the compounds relating the distribution coefficients of the MIP and NIP of each compound [45] using the equation

(4) $$I=\frac{K_{p(MIP)}}{K_{p(NIP)}}$$

Using the previous equation, we can find the specific selectivity factor (S) which relates the impression factors of the MIP and NIP of the template-interfering set [46]:

(5) $$S=\frac{I_{CUR}}{I_{INT}}$$

### 2.6. Real Samples

The adsorption capacity of the MIP was evaluated by analyzing two real samples: turmeric root (obtained in the market) and seasoning. The analysis in real samples consists of a previous stage of standard addition to determine the curcumin content in the turmeric sample to be analyzed. Once the curcumin concentration was obtained, a 20 mg L^−1^ methanol:buffer pH 6 (1:1) solution was prepared. In this solution, 2 mg of MIP was added followed by stirring for 60 min to evaluate the adsorption using UV-visible spectrophotometry.

## 3. Results

### 3.1. Characterization Experiments

**Figure 3:**
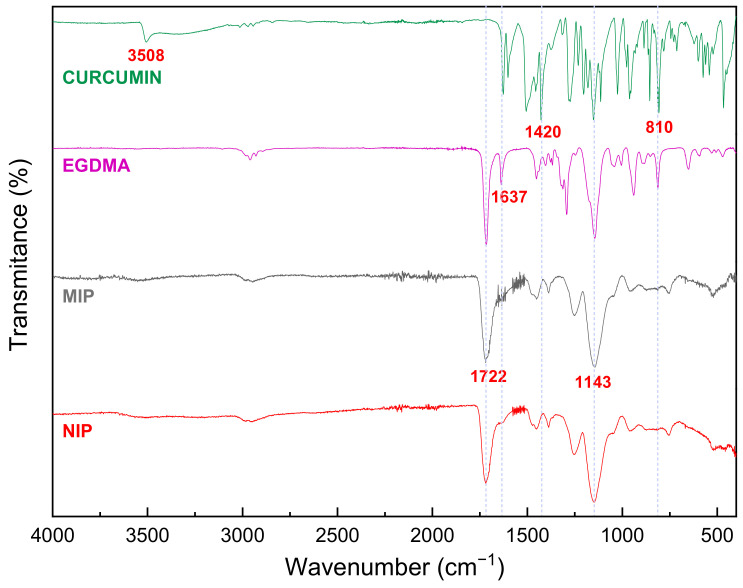
FTIR spectra of Curcumin, EGDMA, MIP, and NIP after being washed using Soxhlet.

The FTIR spectrum of curcumin (Figure 3) shows characteristic peaks in 3508 cm^−1^ corresponding to the stretching vibration of the phenolic OH, 1637 cm^−1^ of the C=C stretch of the aromatic ring, 1597 cm^−1^ from the stretch of the aromatic ring, 1509 cm^−1^ from the stretching vibrations of the C=O and C=C of the chain, 1278 cm^−1^ of the stretching vibration of the C-O of the aromatic carbon, and 1024 cm^−1^ from the stretching vibration of the C-O-C bond [47]. The characteristic bands at 810 cm^−1^ assigned to the vibration of the C-H of the benzene ring and at 1420 cm^−1^ associated with the stretching of the aromatic bond C=C, present in the curcumin spectrum but not in the spectra of polymers indicates the absence of curcumin on the surface of the MIP after washing [48]. 

Also, we can note the FTIR spectra of the MIP and NIP and their similarities with the structural monomer EGDMA: 1722 cm^−1^ corresponding to bond C=O and 1143 cm^−1^ corresponding to the bond C-O; both bonds are from the ester group of structural monomers. These bands are predominant in the spectra of the polymers since they are mainly composed of EGDMA in their structure. The decrease in the intensity of the band at 1637 cm^−1^ of C=C is the product of the polymerization reaction since the double bond is broken [49]. Figure S1 also shows the similarities between MIP and NIP.

**Figure 4:**
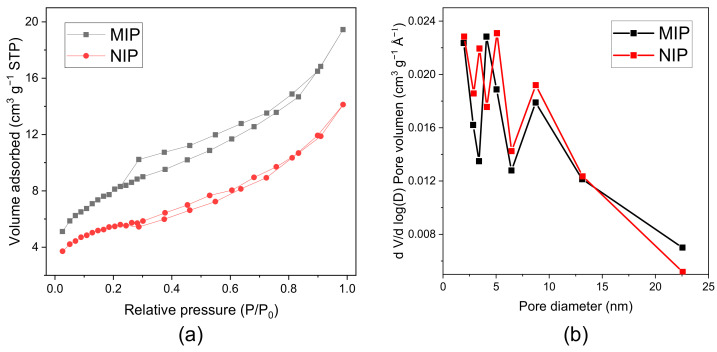
(**a**) N_2_ adsorption–desorption isotherms using the BET method; (**b**) Pore size distribution using the BJH method for polymers MIP y NIP.

The BET isotherms of MIP and NIP are of type IV (Figure 4a), characteristic of micro-mesoporous solids. In addition, due to the phenomenon of capillary condensation, both presented hysteresis, this being the H3 type with parallel plate and wedge-shaped pores [50].

The results obtained from the BET analysis are presented in Table 2 where we highlight a greater BET surface area of the MIP (28.5 m^2^ g^−1^) than of the NIP. (18.5 m^2^ g^−1^). Likewise, the MIP presented a high mesoporous area, characteristic of polymers with pore diameters between 2 and 50 nm (Figure 4b) that adsorb larger molecules such as curcumin [51]; this will be reflected in the greater adsorption capacity of the MIP.

### 3.2. Curcumin Degradation Assays

**Figure 5:**
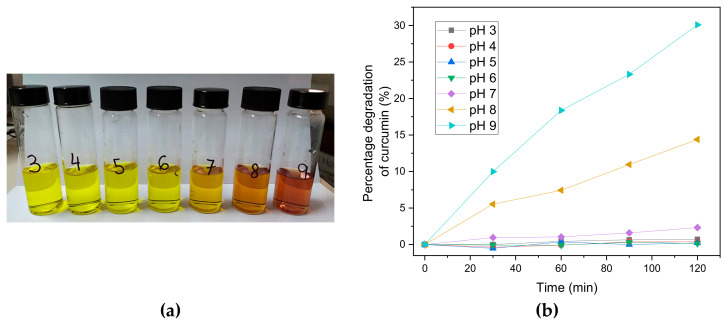
(**a**) Curcumin solutions of 20 mg L^−1^ at different pH values from 3 to 9; (**b**) Curcumin degradation test.

We will analyze the stability of curcumin at different pHs through degradation tests. For this, solutions of 20 mg L^−1^ of curcumin were used in methanol:buffer (1:1) at different pHs (Figure 5a).

The results showed that the possible working range is at pH 2, 3, 4, 5, 6, and 7 (Figure 5b). This result confirms that the enol form is chemically more labile than the keto form, which explains the poor chemical stability of curcumin in basic solutions [52].

### 3.3. UV-Visible Analysis of Curcumin

For curcumin analysis, absorbance spectra were performed in the visible range from 200 to 700 nm. The medium used was a mixture of methanol: buffer pH 6 (1:1), where the solutions presented a bright yellow coloration corresponding to the main intense absorption band at 430 nm that is associated with the low energy π–π* excitation of the chromophore [53].

**Figure 6:**
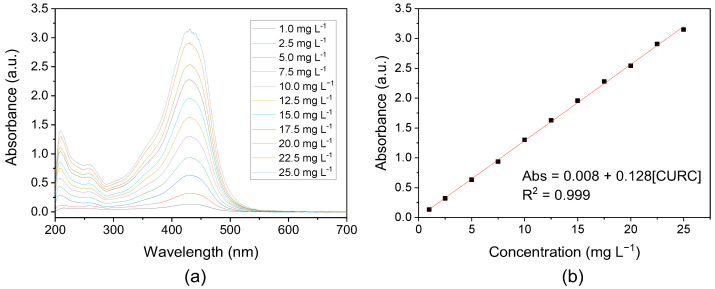
(**a**) Absorbance spectrum of curcumin in methanol:buffer (1:1) pH 6 solution; (**b**) Calibration curve at 430 nm.

The calibration curve obtained in the concentration range of 1 to 25 mg L^−1^ shows linearity with a correlation coefficient of 0.999 (Figure 6).

### 3.4. Optimization of MIP Adsorption

**Figure 7:**
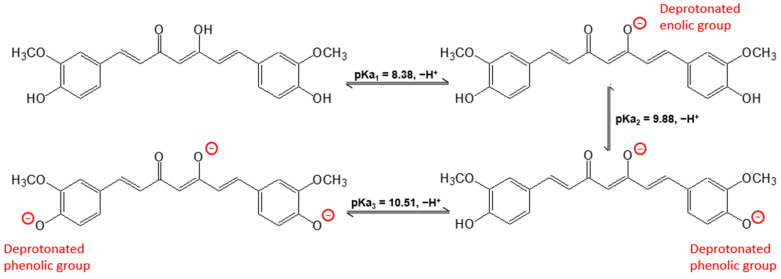
Curcumin structures at different pH values [55].

**Figure 8:**
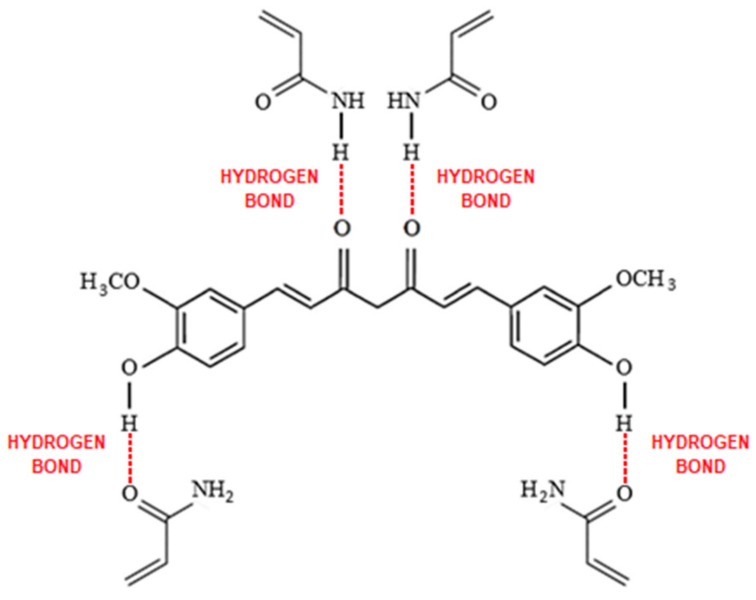
Noncovalent interaction between curcumin–acrylamide.

Curcumin presents three different pKa values that come from the dissociation of enol form (pKa_1_ = 8.38) and OH phenolics groups (pKa_2_ = 9.88 and pKa_3_ = 10.51) (Figure 7) [54]. Based on its structure, we can predict the types of interactions present between curcumin and MIP in the adsorption step. The interactions between acrylamide and curcumin can be of the noncovalent type due to the presence of nitrogen and oxygen (both of high electronegativity), which can act as hydrogen acceptors in acrylamide, forming hydrogen bonds with the phenol groups and the diketonic group of curcumin (Figure 8).

**Figure 9:**
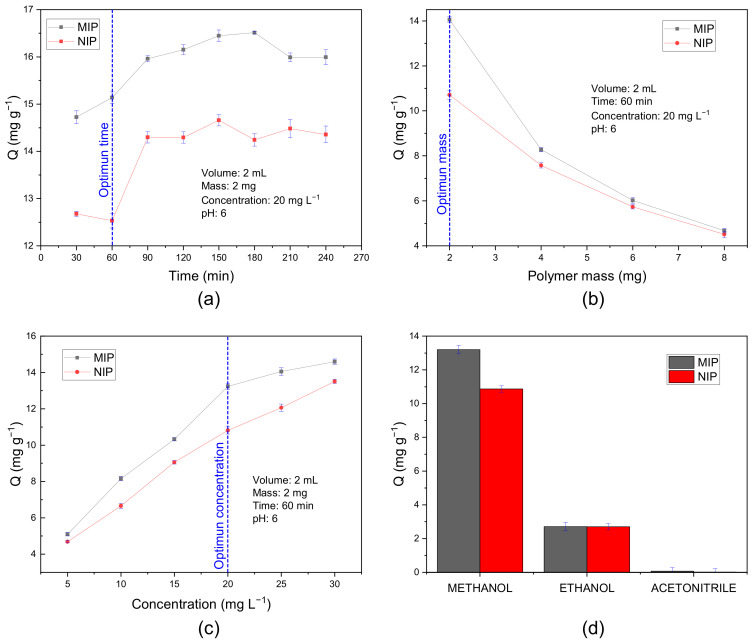
Study of adsorption to optimize different parameters: (**a**) Interaction time; (**b**) Polymer mass; (**c**) Curcumin concentration; (**d**) Adsorption solvent.

The adsorption capacity of the MIP increases linearly up to 90 min, after which time it remains constant. A greater difference between MIP and NIP is obtained in 60 min (Figure 9a). Similarly, the adsorption capacity increases along with the concentration of the curcumin solution, reaching a greater difference in MIP-NIP at 20 mg L^−1^ (Figure 9b). On the other hand, as the mass of the polymer increases, the adsorption capacity decreases. From 4 mg the difference between these is reduced six times less compared to 2 mg (Figure 9c). 

The response of the polymers against polar organic solvents was also evaluated. For this purpose, mixtures of methanol, ethanol, and acetonitrile with pH 6 buffer in a 1:1 ratio were used. The interaction of curcumin against the solvents ethanol and methanol is a hydrogen bond type, unlike the lower dipole-dipole interaction against acetonitrile. The adsorption capacity of the MIP in each solvent can be affected by the competition between the solvent and the analyte for the MIP recognition sites. We noticed that there is a significant difference in the adsorption capacity using the methanol mixture compared to the other two solutions (Figure 9d). The higher polarity of methanol versus ethanol will increase the polarity of the curcumin solution and with it its affinity for the MIP being an important factor due to its higher adsorption capacity.

### 3.5. Adsorption Isotherm and Kinetic

The evaluation of the chemical kinetics is important to obtain information on the control of the speed and the mechanism of the union in the adsorption. For this, the previous data obtained in the polymer optimization tests are adjusted pseudo-first-order and pseudo-second-order kinetic models [56].

Table 3 shows the values of the rate constants and R^2^ for the MIP and NIP. The value of R^2^ for pseudo-second-order kinetics (R^2^ > 0.999) is higher than for pseudo-first-order; on the other hand, the value of Qe calculated, following pseudo-second-order-kinetics, was 16.353 mg g^−1^, which is close to the experimental value (Qe = 16.441 mg g^−1^). Therefore, the curcumin adsorption process by MIP follows pseudo-second-order kinetics, indicating that the process is controlled mainly by chemical action (sharing and transfer of electrons between the adsorbent and adsorbate) and not by the process of mass transfer [57].

We also evaluated the adsorption capacity of MIP and NIP using the Langmuir and Freundlich adsorption models. The linear adjustments obtained (Table 4) show R^2^ values greater than 0.98 following the Freundlich model, in addition to the value of 1/n less than 1, which indicates strong interactions between adsorbent–adsorbate and is attributed to the heterogeneity of adsorption sites. The MIP also presented a higher value of the Kf constant, this indicates its strong affinity towards curcumin. In this way, the curcumin adsorption results better fit the Freundlich isotherm model, associated with noncovalent adsorption characteristic of MIPs in heterogeneous systems [57,58].

### 3.6. Selectivity

**Figure 10:**
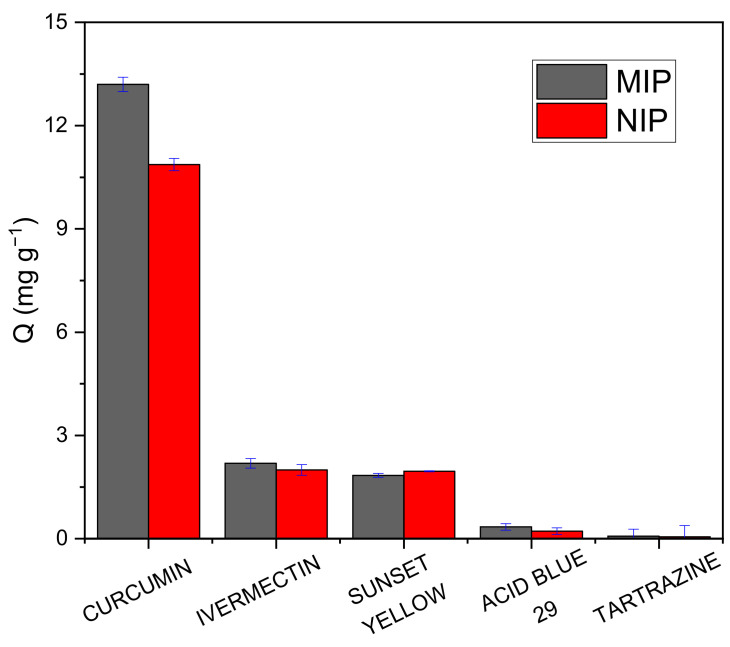
Selectivity study against interferents ivermectin, sunset yellow, acid blue 29, and tartrazine employing optimum parameters. The measurements were made at the maximum absorbance wavelengths of each interferent.

The selectivity study allows us to identify the correct formation of selective cavities in the MIP from the analysis of the adsorption capacity values (Figure 10); for this reason, we analyzed the impression factor (I), which turns out to be higher for curcumin which indicates that MIP has higher molecular recognition of curcumin with respect to NIP. The specific selectivity factor (S) obtained is greater than 1 in all cases (Table 5), which indicates the molecular memory of the MIP for curcumin, its interactions, and size [45].

### 3.7. Real Sample

MIP was used to determine the amount of curcumin absorbed from turmeric root and seasoning samples at the optimal parameters previously obtained. Table 6 shows the ability of the MIP to quantify curcumin and recovery percentage greater than 70%.

## 4. Conclusions

This study for the first time reported the development of molecular imprinting polymers (MIP) capable of selective detection of curcumin in real food samples. The optimal adsorption parameters obtained using UV-visible spectrophotometry were as follows: 60 min of interaction time, 2 mg of adsorbent mass, 20 mg L^−1^ adsorbate concentration, and a mixture of methanol: buffer pH 6 as an adsorption medium. Likewise, the selectivity parameters obtained for the MIP were 15.8 as separation factor (α), 1.63 as impression factor (I), and 1.56 as specific selectivity factor, achieving the selectivity of the MIP against various interferents. The analyses carried out on real turmeric samples gave the adsorption results of 15.82 mg g^−1^ and 14.09 mg g^−1^ for the root and seasoning samples, respectively. The recovery percentages obtained in the quantification of curcumin were greater than 70% on average, high results for a less sophisticated method compared to the methods indicated in Table 1 where the recovery percentages are greater than 80%. The improvement of these polymers can be carried out using nano-MIPs since it would facilitate the removal stage of the template, generating more homogeneous spherical particles and better-defined cavities. Similarly, MIP can be immobilized on transducer surfaces such as fiber optics to form an optical sensor, improving its detection ability, selectivity, and sensitivity.

## Figures and Tables

**Table 1:** Comparative study of the fabricated material with the literature for Curcumin.

Material	Analyte/ Real Sample	Method	LOD/ % Recovery	Ref.
Magnetic MIP	Curcumin/ Curry and ginger	UV-vis	1.31 µg mL^−1^ 79.37~88.89%	[18]
MIP-SPE	Curcuminoid/ Pharmaceutical and urine sample	HPLC with fluorescence detection	5.0 mg L^−1^ >80.00%	[40]
Magnetic MWCNT/MIP	Curcumin/ Ginger powder and kiwi fruit root	HPLC	99.3~100.5% 95.8~97.2%	[41]
MIP-CPE Sensor	Curcumin/ Curcuma powder and cookies	CV	10.1 nmol L^−1^ 90.77~105.7%	[42]
PAA-MIP/G Sensor	Curcumin/ Turmeric powder and capsules	DPV	0.04 µmol L^−1^ >99.00%	[16]
MIP	Curcumin/ Curcuma root and powder	UV-vis	0.699 mg L^−1^ 70.5~79.1%	This work

**Table 2:** BET surface area and porosity obtained for the MIP and NIP polymers.

Polymer	BET Surface Area (m^2^ g^−1^)	Micropore Area (m^2^ g^−1^)	Mesopore Area (m^2^ g^−1^)	Average Pore Diameter (nm)
MIP	28.5	0.7	27.8	4.3
NIP	18.5	6.2	12.3	4.6

**Table 3:** Parameters obtained following kinetics models of pseudo-first-order (A) and pseudo-second-order (B).

Kinetic Model	Polymer	R^2^	Q_e_ (mg g^−1^)	Kinetic Constant (K) (min^−1^)
(A) Pseudo-first- order	MIP	0.9590	3.631	0.0209
	NIP	0.7810	4.786	0.0228
(B) Pseudo-second-order	MIP	0.9987	16.353	0.0252
	NIP	0.9984	14.848	0.0075

**Table 4:** Data obtained using the Langmuir (**A**) and Freundlich (**B**) isotherm models applied to the experimental data.

Polymer	(A) Langmuir Isotherms Parameters		
	R^2^	Qmáx (mg g^−1^)	K_L_ (L mg^−1^)
MIP	0.9794	24.68	0.0511
NIP	0.9676	22.82	0.0456
**Polymer**	**(B) Freundlich Isotherms Parameters**		
	**R^2^**	**1/n**	**Kf (mg g^−1^) (L mg^−1^)^1/n^**
MIP	0.9850	0.6102	1.9676
NIP	0.9959	0.6029	1.7415

**Table 5:** Selectivity parameters for the adsorption of interferents by the MIP and NIP, using each compound at a concentration of 20 mg L^−1^.

Interferent	Q-MIP (mg g^−1^)	Q-NIP (mg g^−1^)	Kp-MIP (mL g^−1^)	Kp-MIP (mL g^−1^)	I	S
Curcumin	13.20	10.87	1.94	1.19	1.63	-
Ivermectin	2.19	2.00	0.12	0.10	1.23	1.33
Sunset Yellow	1.84	1.96	0.10	0.10	0.98	1.67
Acid Blue 29	0.22	0.34	0.01	0.02	1.56	1.04
Tartrazine	0.08	0.06	0.004	0.003	1.34	1.22

**Table 6:** Results obtained using two samples from markets in the city of Lima (Lima Region, Peru).

Real Sample	% Curcumin	Q_ADS_ (mg g^−1^)	% Recovery
Turmeric root	2.6	15.82	79.1
Seasoning stick	1.1	14.09	70.5

## Data Availability

Not applicable.

## Supplementary Materials

The following supporting information can be downloaded at: https://www.mdpi.com/article/10.3390/polym15163332/s1, Figure S1: Thermogravimetric analysis (TGA) curves obtained for MIP and NIP; Table S1: Effect of the type of functional monomer, type of crosslinker and ratios of curcumin functional monomer—crosslinker used in the preparation of MIP and NIP.
