# Supplementary material for: Synthesis, Characterization, and Evaluation of a Novel Molecularly Imprinted Polymer (MIP) for Selective Quantification of Curcumin in Real Food Sample by UV-Vis Spectrophotometry
Source: Polymers (Basel). 2023 Aug 8;15(16):3332. doi: 10.3390/polym15163332 (PMC10458492; doi:10.3390/polym15163332)
Supplement: Supplementary file 1 [file polymers-15-03332-s001.zip › polymers-2525627-supplementary.pdf]

polymers

MDPI

Supplementary Materials

Synthesis, Characterization, and Evaluation of a Novel Molecularly Imprinted Polymer (MIP) for Selective Quantification of Curcumin in Real Food Sample by UV-Vis Spectrophotometry

Sergio Espinoza-Torres [1], Rosario López [1], Maria D. P. T. Sotomayor [2], Juan C. Tuesta [3], Gino Picasso [1] and Sabir Khan [1,2,4,*]

[1] Technology of Materials for Environmental Remediation Group (TecMARA), Faculty of Sciences, National University of Engineering, Av. Tupac Amaru 210, Rimac 15333, Peru; sespinozat@uni.pe (S.E.-T.); rclopez@uni.edu.pe (R.L.); gpicasso@uni.edu.pe (G.P.)

[2] Chemistry Institute, São Paulo State University (UNESP), Araraquara 14801-900, Brazil; m.sotomayor@unesp.br

[3] Laboratorio de Biotecnología, Universidad Nacional Autónoma de Alto Amazonas, Calle Prolongación Libertad 1220, Yurimaguas 16501, Peru; jtuesta@unaaa.edu.pe

[4] Department of Natural Sciences, Mathematics, and Statistics, Federal Rural University of the Semi-Arid, Mossoro 59625-900, Brazil

* Correspondence: sabir@ufersa.edu.br or skhan@uni.edu.pe

SI.1. Thermogravimetric analysis

TGA curves obtained for MIP and NIP (figure S1) showed similar behaviour. At temperatures below 240 °C, the polymers presented a mass loss of less than 5% because of solvent residues. On the other hand, in the range from 240 °C to 450 °C the mass loss reaches 90%, this temperature corresponds to the decomposition of the crosslinking agent (EGDMA) and, to a lesser extent, to acrylamide, with a temperature slightly higher (250 °C) for MIP due to the presence of some curcumin molecules that increase the decomposition temperature of this polymer, thus increasing its stability.

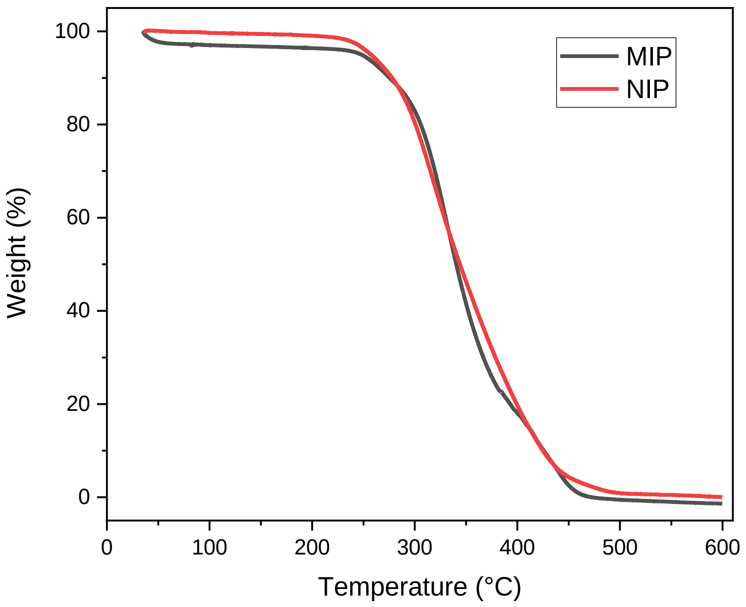

Figure S1. Thermogravimetric analysis (TGA) curves obtained for MIP and NIP.

SI.2. Evaluation of components for the preparation of MIP and NIP

Table S1. Effect of the type of functional monomer, type of crosslinker and ratios of curcumin functional monomer – crosslinker used in the preparation of MIP and NIP.

Polymer code	Functional monomer (mmol)		Curcumin (mmol)	Crosslinker (mmol)		Initiator ABCV (g)	Porogenic solvent Acetonitrile (mL)
	AM	MAA		EGDMA	TRIM		
**AM_1**	**0.2**	**-**	0.05	1.0	-	0.1	20
**AM_2**	**0.2**	**-**	**0.05**	**2.5**	**-**	**0.1**	**20**
AM_3	0.2	-	0.05	5.0	-	0.1	20
MAA_1	-	0.2	0.05	1.0	-	0.1	20
MAA_2	-	0.2	0.05	2.5	-	0.1	20
MAA_3	-	0.2	0.05	5.0	-	0.1	20
AM_4	0.2	-	0.05	-	2.5	0.1	20
MAA_4	-	0.2	0.05	-	2.5	0.1	20
